# What is in your cup of tea? *DNA Verity Test* to characterize black and green commercial teas

**DOI:** 10.1371/journal.pone.0178262

**Published:** 2017-05-23

**Authors:** Olga De Castro, Maria Comparone, Antonietta Di Maio, Emanuele Del Guacchio, Bruno Menale, Jacopo Troisi, Francesco Aliberti, Marco Trifuoggi, Marco Guida

**Affiliations:** 1 Department of Biology, Botanical Garden, University of Naples Federico II, Naples Italy; 2 Botanical Garden, University of Naples Federico II, Naples Italy; 3 Department of Medicine and Surgery and Dentistry, University of Salerno, Salerno, Italy; 4 Department of Biology, University of Naples Federico II, Naples Italy; 5 Department of Chemical Sciences, University of Naples Federico II, Naples, Italy; Universita degli Studi di Milano-Bicocca, ITALY

## Abstract

In this study, we used several molecular techniques to develop a fast and reliable protocol (*DNA Verity Test*, *DVT*) for the characterization and confirmation of the species or taxa present in herbal infusions. As a model plant for this protocol, *Camellia sinensis*, a traditional tea plant, was selected due to the following reasons: its historical popularity as a (healthy) beverage, its high selling value, the importation of barely recognizable raw product (i.e., crushed), and the scarcity of studies concerning adulterants or contamination. The *DNA Verity Test* includes both the sequencing of DNA barcoding markers and genotyping of labeled-PCR DNA barcoding fragments for each sample analyzed. This protocol (*DVT*) was successively applied to verify the authenticity of 32 commercial teas (simple or admixture), and the main results can be summarized as follows: (1) the *DVT* protocol is suitable to detect adulteration in tea matrices (contaminations or absence of certified ingredients), and the method can be exported for the study of other similar systems; (2) based on the BLAST analysis of the sequences of *rbc*L+*mat*K±*rps*7-*trn*V^(GAC)^ chloroplast markers, *C*. *sinensis* can be taxonomically characterized; (3) *rps*7-*trn*V^(GAC)^ can be employed to discriminate *C*. *sinensis* from *C*. *pubicosta*; (4) ITS2 is not an ideal DNA barcode for tea samples, reflecting potential incomplete lineage sorting and hybridization/introgression phenomena in *C*. *sinensis* taxa; (5) the genotyping approach is an easy, inexpensive and rapid pre-screening method to detect anomalies in the tea templates using the *trn*H^(GUG)^-*psb*A barcoding marker; (6) two herbal companies provided no authentic products with a contaminant or without some of the listed ingredients; and (7) the leaf matrices present in some teabags could be constituted using an admixture of different *C*. *sinensis* haplotypes and/or allied species (*C*. *pubicosta*).

## Introduction

An ancient English proverb states “*The path to heaven passes through a teapot*”, and this proverb is sadly true in recent years. In fact, considering the latest dramatic news concerning food fraud and soil contamination (e.g., soil contamination in Indian tea estates soil; [1, 2]), it is likely that “*the path to heaven*” can be accelerated by what you drink or eat (e.g., the tragic case of the “*soil of fire*” in southern Italy; [3, 4]). Currently, the adulteration of food is frequent, and the scientific community has used been advanced technologies to protect humans from incorrect practices in food or drink production (e.g., [5, 6, 7, 8, 9, 10]). Indeed, some products can represent real and dangerous “mirror for larks” in terms of health, as the presumed natural or biological aliments or supplements purchased by consumers to protect their health lack valid quality controls [11]. Moreover, these controls are occasionally difficult to perform, reflecting the intrinsic nature of the product (e.g., food supplements or herbal medicine [12, 13, 14]). The international trade of herbal products is in continuous development for both alimentary and pharmaceutical purposes [15]; in fact, many plants are used daily in the preparation of foods and herbal teas (e.g., [16, 17]). Specifically, tea infusions are widely used as both pleasant drinks and for their many beneficial properties [18], and an accurate definition of the compounds present in these teas is important not only for the consumer but also for the producers and control authorities [19]. Because of the fragmentation or pulverizing of the vegetal material, it is often difficult to identify the species among the ingredients using traditional analyses (i.e., macroscopic and microscopic morphology) [20, 21]. Thus, a DNA barcoding technique has recently been used to address this problem, resulting in a useful system for the detection of the plants employed in these teas and the characterization of the possible adulteration and/or contamination in a wide range of plant-based foods (e.g., [22, 23]), becoming a universal adopted approach in the last few years [24, 25].

In this study, we used a multi-faceted DNA barcoding approach to develop a fast and reliable protocol (*DNA Verity Test*, *DVT*) for the taxonomic characterization and confirmation of herbal infusions. As a model plant for this protocol, *Camellia sinensis* (L.) Kuntze, the traditional tea plant, was selected for (1) its historical popularity as a (healthy) beverage [26, 27, 28], (2) its high selling value (cfr. [27]), (3) the importation of barely recognizable raw product (i.e., crushed) [29], and (4) the scarcity of studies concerning adulterants or contaminations [30, 31].

The *DNA Verity Test* was successively applied to verify the authenticity of 32 commercial tea packages (simple or admixture). Briefly, the *DNA Verity Test* verified both the sequencing of DNA barcoding markers and the genotyping of labeled-PCR DNA barcoding fragments for each sample analyzed.

The methodology of *DNA Verity Test* presented here is very detailed and is a promising tool for checking the authenticity of tea samples and it could be also suitable for application in different study systems.

In addition, the development of *DNA Verity Test* is part of a wider project comprising a multi-faceted pilot study with the purpose of analyzing 32 brands of European and Italian commercially available teas (16 black and 16 green teas) using different analytical approaches evaluate the presence of mycotoxins, microbial contaminants, heavy metals and phthalates (O. De Castro, unpublished data).

## Materials and methods

### Tea sampling design

Currently, tea products are available at a variety of mainstream outlets, such as supermarkets, health food stores, drug stores and online from herbal supply companies. In the present study, a total of 32 tea packages (*C*. *sinensis*) were purchased from markets in Naples (southern Italy) and from online-shops (Table 1) and subsequently tested using a blind experiment for the analyses reported below. All products were also available to consumers through online-shops, representing 17 Italian or internationally famous brands (seven and ten, respectively). Within these 17 brands, 16 samples were fermented tea (only black tea) and 16 samples were raw tea (only green tea). Decaffeinated, soluble, admixture (i.e., the presence of other plant material) and flavored samples were also considered (Table 1). The tea samples were selected considering the trade network (supermarket, drug store and herbalist shop), the price (cheap and expensive), the marketing quality (packaging, publicity and brand) and the presence of filters in the packages (except for one type of soluble green tea). This information (except the brand name) is reported in Table 1. The tea samples were stored at room temperature prior to the molecular analyses, and duplicates for each tea package-lot were analyzed. As a preliminary molecular analysis, specimens of *C*. *sinensis* (CS-DNAs) were obtained from the Botanical Garden of Naples (BGN). The tissues of other species indicated in the admixture tea samples were obtained from BGN as reference samples {i.e., *Mentha* L. sp. (peppermint and corn mint), *Citrus* L. sp. (lemon, orange, grapefruit, and lime), *Pimpinella anisum* L. (anise), *Cinnamomum verum* J. Presl or *C*. *cassia* (L.) J. Presl (cinnamon), and *Glycyrrhiza glabra* L. (licorice)}.

**Table 1:** List of Italian commercialized black and green tea packages analyzed in the present study (N and V samples, respectively). Information for each accession about the marketing quality (high, medium and low), sales network (D = discount supermarket; H = herbalist shop; S = supermarket; P = drugstore), price (ϵ) {(A), < 1 ϵ; (B), 1 < ϵ < 2; (C), 2 < ϵ < 4; (D), 4 < ϵ < 6; (E), > 6 ϵ} and molecular results for *rbc*L and *rps*7-*trn*V^(GAC)^ sequences (presence of a SNP in the 68 bp coding region of *rbc*L, A = adenine, C = cytosine; *rps*7-*trn*V^(GAC)^, 239 bp = *Camellia sinensis*, 226 bp = *C*. *pubicosta*; in smaller font, the nucleotide/fragment less represented).

Code	Marketing quality*	Sales network	Price	Labelling information	*rbc*L haplotype (68 bp SNP)	*rps*7-*trn*V^(GAC)^ haplotype (bp)
**Black teas**						
**N1**	Medium	H	D	Organic black tea leaves of *Camelia sinensis* (L.) Kuntze (100%)	A/c	239
**N2**	Medium-good	P, H	D	Tea leaves [*Camellia sinensis* (L.) Kuntze]	C	239
**N3**	Good	S	B	Black tea, aromas	C	239
**N4**	Low-medium	S	C	Not reported	C	239
**N5**	Good	P, H	C	Black certified tea (100%)	C/a	239
**N6**	Good	S	B	Tea	C	239/_226_
**N7**	Good	S	C	Decaffeinated tea	C	239
**N8**	Good	S	B	Not reported	C	239/_226_
**N9**	Good	S	C	Black tea (94%), lemon aroma (6%)	C/a	239/226
**N10**	Low	S	B	Black tea (100%)	C	239/_226_
**N11**^†^	Good	S	D	Decaffeinated black tea, caffeine < 0.1%	C/a	239/_226_
**N12**	Low	S	B	Black tea	C	239/_226_
**N13**	Low	S	B	Black tea (95%), natural lemon aroma (5%)	C/a	239/_226_
**N14**	Low	S	B	Black tea leaves	C	239/_226_
**N15**	Medium	H	C	Decaffeinated organic black tea, caffeine < 0.1%	C	239
**N16**	Good	S	C	Sugar, acidifier (citric acid), decaffeinated tea extract, aromas, lemon juice powdered (0.5%); gluten free	N.d.	N.d.
**Green teas**						
**V1**	Good	P, H	E	Pure leaves of green tea (*Camelia sinensis* Kuntze)	A	239
**V2**	Medium	P, H	E	Green tea leaves (*Camelia sinensis*) (99%), bergamot essential oil (1%)	A	239
**V3**	Medium	H	D	Organic green tea leaves [*Camellia sinensis* (L.) Kuntze] (100%)	A	239
**V4**	Medium	P, H	D	Green tea leave	A	239
**V5**	Medium-good	P, H	D	Unfermented organic tea leaves (*Camellia sinensis* L.)	A/c	239
**V6**	Medium	H	C	Green tea certified by Fairtrade	C	239/_226_
**V7**	Medium	H	D	Decaffeinated; ingredient not listed	A	239
**V8**	Good	S	C	Green tea (100%)	C	239/_226_
**V9**	Good	S	C	Green tea, aromas, and citrus peels (2.1%: grapefruit, lemon, lime, and orange)	C	239/226
**V10**^†^	Good	S	C	Green tea, aromas, and spices (2%) (anise, cinnamon, and licorice)	C	239/226
**V11**	Good	S	C	Green tea, aromas, and mint (7.9%)	C	239/226
**V12**	Good	S	C	Green tea	A/c	239
**V13**	Low	S	B	Green tea	A	239
**V14**	Low	S	B	Organic green tea	A/C	239
**V15**	Low	D	A	Green tea	A/C	239
**V16**	Medium	S	A	Sugar, acidifier (citric acid), green tea soluble extract, aromas, and ginseng soluble extract	N.d.	N.d.

*, value determined by a questionnaire conducted on 25 people (13 females and 12 males)

^**†**^, adulterated product; N.d., no datum.

### DNA Verity Test (DVT) procedure

#### Barcode markers

Candidate DNA barcoding markers were selected based on previous tests, which had been conducted using universal primers [25, 31, 32, 33, 34], GenBank data and reference *C*. *sinensis* DNA from BGN. The following four criteria for selecting an ideal nucleotide sequences barcode were considered: (1) highly efficient amplification, (2) high quality sequences (e.g. no unambiguous sequences caused by double peaks or stuttering effect), (3) an exhaustive sequence database publicly available, and (4) high species discrimination capability.

The following two molecular techniques were used for the *DNA Verity Test* (*DVT*): sequencing via Sanger chemistry and genotyping of fluoresced amplified fragment via capillary electrophoresis. After in silico and laboratory tests, the candidate markers for the sequencing approach included the following: genes and plastid intergenic spacers {*rbc*L, *mat*K and *trn*H^(GUG)^-*psb*A, *rps*7-*trn*V^(GAC)^, respectively} and a nuclear intergenic spacer (ITS2). For the genotyping analyses, the considered markers were: an intron {P6 loop of the *trn*L^(UAA)^}, plastid intergenic spacers {*trn*H^(GUG)^-*psb*A and *rps*7-*trn*V^(GAC)^}, and a nuclear intergenic spacer (ITS2). The universal barcode primers and the specific barcoding primers designed in the present study are reported in Table 2.

**Table 2:** Primers used in the present study for DNA barcoding analyses.

Locus	Primer name	Sequence (5’-3’)	Ta	Primer note	Reference
***rbc*L**	rbcLa_F	ATG TCA CCA CAA ACA GAG ACT AAA GC	55	Universal	[35]
	rbcLajf634R	GAA ACG GTC TCT CCA ACG CAT		Universal	[36]
	CS_rbcL-300rev	TAA AGG ATA CGC TAC ATA AGC	55	Specific for *Camellia sinensis*; used to check the SNP-M (68 bp) in sequencing PCR	In the present study
***mat*K**	1R_KIM-f	ACC CAG TCC ATC TGG AAA TCT TGG TTC	55	Universal	Ki-Joong Kim, pers. comm.
	3F_KIM-r	CGT ACA GTA CTT TTG TGT TTA CGA G			
***trn*H**^**(GUG)**^**-*psb*A**	psbA3'f	GTT ATG CAT GAA CGT AAT GCT C	55	Universal	[37]
	trnHf*	GTT ATG CAT GAA CGT AAT GCT C			[38]
	Cinnamon_2psb-for	ACG AGT CGT TGA AGG ATC AAT	56	Specific for *Cinnamomum verum* and *C*. *cassia* (cinnamon)	In this study
	Cinnamon_2trnH-rev	TGC AGG TTG GTA CAG AAG AA			
	Citrus_psbA-in	GTA TTG ATC CGT TAT TTA GTC G	57	Specific for *Citrus* sp. (lemon, orange, grapefruit, and lime)	In this study
	Citrus_trnH-in	ATC TTA TCT TAC TTA TGA AGA ACC			
	Glycyrrhiza_psbA-in	GTT TTA AAG AAG GAT ACG AGG	55	Specific for *Glycyrrhiza glabra* (licorice)	In this study
	Glycyrrhiza_trnH-inA	TAC ATT CGC CCT TCT TAT AC			
	Mentha_2psb_for	TTC CAG GCA AGT CCA ATA CT	56	Specific for *Mentha* sp. (*M*. *aquatica*, *M*. *arvensis*, *M*. *canadensis*, *M*. *longifolia*, *P*. *pulegium*, *M*. *suaveolens*, *M*. *pulegium*, and *M*. x *piperita*, *M*. *spicata*)	In this study
	Mentha_2trnH_rev	TGT GTA GGA GTT TTT GAA AAT AGA C			
	Piminella_psbA-in	ACC TAG CTG CTG TTG AAG CTC	60	Specific for *Pimpinella anisum* (anise)	In this study
	Pimpinella_trnH-in	GGA GCA ATA TCG CTT TCT TGA TAG A			
***rps*7-*trn*V**^**(GAC)**^	CS_*rps*7_1	GTG CTA TGG CTC GAA TCC GT	67	Specific for *Camellia* sp.; used to discriminate *C*. *sinensis* from *C*. *pubicosta*	In this study
	CS_*trn*V^(GAC)^_1*	ACC ACG TCA AGG TGA CAC TC			
**P6 loop *trn*L**^**(UAA)**^	g	GGG CAA TCC TGA GCC AA	55	Universal	[39]
	h*	CCA TTG AGT CTC TGC ACC TAT C			
**ITS2**	S2F	ATG CGA TAC TTG GTG TGA AT	55	Universal	[40]
	S3R*	GAC GCT TCT CCA GAC TAC AAT			
	BELL-1	GGD GCG GAK AHT GGC CYC CCG TGC	55	Universal	[41]
	BELL-3	GAC GCT TCT CCA GAC TAC AAT			

*, labeled

#### Tea DNA extraction

A total of 30 mg of shredded material present in tea bags from each package was used for DNA extraction. Aiming at selecting the best DNA isolation procedure to obtain high yield and quality of extracted DNA [42], a preliminary analysis on three samples (green, black and admixture tea) was performed using four commercial kits {PowerPlant Pro DNA Isolation Kit (Mo Bio), Plant Genomic DNA Extract Mini Kit (Fisher Molecular Biology), ZR Plant/Seed DNA MicroPrep (Zymo Research), GeneAll Exgene Plant SV kit (GeneAll Biotechnology)} and two detergent protocols [43, 44]. The isolated DNA was analyzed using both a spectrophotometer Nanodrop 2000 (Thermo Fisher Scientific) to quantify its purity grade (260/280 and 260/230) and a Qubit 3 Fluorometer to determine the precise DNA concentration (Life Technologies, Thermo Fisher Scientific). In addition, a visual estimate was obtained using 1% agarose electrophoresis with Gel Red strained (Biotium) band intensities and GeneRuler 1 kb Plus DNA Ladder (Thermo Fisher Scientific). After preliminary analysis, all extracted genomic DNA were estimated using both fluorometric and electrophoresis analyses.

For fresh plant references from BGN, 100 mg of leaf tissue was used for the DNA extraction using GeneAll Exgene Plant SV kit (GeneAll Biotechnology) according to the manufacturer's instructions.

#### PCR amplification

Molecular markers were amplified using a high-fidelity DNA polymerase, and the primers are listed in Table 2. The PCRs were performed using 10 ng of genomic DNA, 5 X Phusion HF buffer and 0.5 uM of each primer with Phusion Hot Start II DNA Polymerase (Thermo Fisher Scientific) according to the manufacturer’s instructions. The amplification of the recalcitrant DNA templates was performed using AmpONE Fast Pfu DNA polymerase (GeneAll Biotechnology).

#### Sequencing approach

According to the sequencing approach, the amplified fragments longer than 350 bp (e.g., *rbc*L/*mat*K) were purified using PEG8000 precipitation (PEG 20%, 2.5 M NaCl). In contrast, polymorphic PCR fragments shorter than 350 bp {i.e., *trn*H^(GUG)^-*psb*A/*rps*7-*trn*V^(GAC)^/ITS2} were purified using Monarch PCR and the DNA Cleanup kit (New England BioLabs). Approximately 10 ng/100 bp of the purified templates were sequenced according to Di Maio and De Castro [45] using a fluorescent dye (Bright Dye Terminator Cycle Sequencing Kit, ICloning). The reactions were purified using BigDye XTerminator Purification Kit (Applied Biosystems, Thermo Fisher Scientific) and read using an automated sequencer (3130 Genetic Analyzer, Life Technologies, Thermo Fisher Scientific). The sequences were analyzed using AB DNA Sequencing Analysis version 5.2 software (Applied Biosystems, Thermo Fisher Scientific Inc.), edited in Chromas lite ver. 2.1.1. software (http://technelysium.com.au/?page_id=13), and assembled and aligned in BioEdit ver. 7.2.5 software [46]. PCR fragments with multiple peaks within the sequence were cloned using the CloneJET PCR Cloning Kit (Thermo Fisher Scientific) according to the manufacturer’s instructions. Transformation was performed using StrataClone SoloPack Competent Cells (Agilent Technologies). The bacteria were cultured in LB medium at 37°C for 30 min and subsequently transferred to LB agar plates containing 100 ug/ml ampicillin. Ninety-six randomly selected clones from each transformation were amplified using the corresponding DNA barcoding primers. For each PCR fragment polymorphic from the *C*. *sinensis* reference (e.g., CS_trnH-PsbA_ ref = 510 bp), five PCR samples were sequenced.

The identification of unknown sequence barcodes from the tea samples was conducted using the Basic Local Alignment Search Tool (BLAST; [47]) implemented in two barcode libraries {GenBank and The Barcode Of Life Data system (BOLD); [48, 49]}. To optimize correct identifications, the closest match for each molecular marker was defined as the target with the highest percentage identity using an arbitrary cut-off of 95% and an E-value < 1e^-4^ or greater in terms of overlap with the query sequence.

#### Genotyping approach

Regarding the genotyping approach, the amplification procedure for the P6 loop, *trn*H^(GUG)^-*psb*A, *rps*7-*trn*V^(GAC)^ and ITS2 markers was the same as that reported above, except for the use of 0.5 uM of fluorescently labeled reverse primer (Table 2). Five microliters {ca. 20 ng (P6 loop), 40 ng (*rps*7-*trn*V^(GAC)^} and 100 ng {*trn*H^(GUG)^-*psb*A and ITS2} of amplified labeled fragments were purified using 2 uL of CleanSweep PCR Purification Reagent (Applied Biosystems, Thermo Fisher Scientific); 0.5 uL {ca. 3 and 7.5 ng, respectively; Relative Fluorescence Units (RFU) > 6000} and 1 uL of 1:5 dilution (ca. 1 and 3 ng, respectively; RFU ≥ 3000) for each purification were loaded onto a 3130 Genetic Analyzer with 0.4 uL of fluorescently labeled internal size standard (GeneScan 1000 ROX or 600 LIZ dye Size Standards, Applied Biosystems, Life Technologies). *Camellia sinensis* PCR-labeled standards were also generated for each marker used (CS-ref) and for the other plant species present in the admixture tea infusions (i.e., mint, lemon, orange, grapefruit, lime, anise, cinnamon and licorice, Table 1). Raw data were scored with an internal size standard using Peak Scanner version 1.0 software (Applied Biosystems, Life Technologies).

## Results and discussion

### DNA Verity Test (DVT) protocol

#### DNA extraction

First, we obtained a standard and rapid method to achieve good quality genomic DNA free from inhibitors (e.g., polysaccharides, polyphenol and phenolic compounds) that could interfere with the activity of DNA polymerase in the PCR amplification, as previously reported [50]. Indeed, according to Graham [51], some compounds are present at high concentrations in the leaves of *C*. *sinensis* (e.g., polysaccharides > 12%, secondary metabolites = 40% on dry leaf weight) and can be co-precipitated with DNA determining failure in PCR amplification reactions. The genomic DNA from the tea samples was isolated using the Exgene Plant SV Kit, which produced a better quantity (70.3 ng/uL ± 4.3 SE) and quality of DNA amplification (A260/A280 = 1.82 ± 0.005 SE; A260/A230 = 1.47 ± 0.02 SE) compared with other kits. The DNA was eluted in 100 uL of nuclease-free molecular biology grade water (Ambion, Thermo Fisher Scientific).

#### PCR amplification

In *C*. *sinensis* reference DNAs (CS-DNAs), *rbc*L oligos produce a fragment of 654 bp; *mat*K oligos produce a fragment of 889 bp; *trn*H^(GUG)^-*psb*A oligos produce a fragment of 510 bp; *rps*7-*trn*V^(GAC)^ produce a fragment of 239 bp; P6 loop oligos produce a fragment of 90 bp and ITS2 oligos produce fragments of 475 bp (ITS2-Chen oligos) and 345 bp (ITS2-Chiou oligos). High efficiency DNA amplification was obtained for all the markers, except for the *mat*K gene region (i.e., low-medium), as also reported in the literature (e.g., [31, 33, 34]). Polymerase stuttering effects were eliminated using high-fidelity DNA Polymerase (Phusion Hot Start II), particularly in *trn*H^(GUG)^-*psb*A, where a fixed polA_13_ was present at the end of sequence {5’-*trn*H^(GUG)^-*psb*A-3’} [52].

#### PCR sequencing

The PCR products from CS-DNAs corresponding to the above DNA markers were sequenced and high-quality sequences were obtained for all markers (i.e., no double peaks caused by more sequences), except for the nuclear markers ITS2-Chen and ITS2-Chiou [40, 41]. Comparing the raw data of electropherograms obtained using the different ITS2 oligos (Chen vs. Chiou), the ITS2-Chious sequences present less noise, but double peaks were consistently present. It is highly likely that the double peaks reflect the different design of the forward primer (BELL1) into a conserved part of ITS2, which amplified a partial ITS2, determining less noise compared with the total ITS2 of Chen *et al*. [40], where the forward oligo is designed before the ITS1 (i.e., 5.8S rRNA). The presence of different ITS2 sequences was also demonstrated through the cloning of the PCR fragment from the reference DNA of two different accessions of *C*. *sinensis*; ten positive colonies for each sample were sequenced and blasted using the GenBank database, confirming the absence of DNA as a contaminant (GenBank accessions: from KY928288 to KY928307). The presence of a multi-family of ITS sequences in the CS-DNAs was also observed in an additional assessment of the tea samples, which did not show contamination phenomena or adulteration detected with the other molecular markers employed. These data (i.e., intra-variability of ITS) were consistent with the literature [42, 53, 54], where a probable incomplete lineage sorting together to a high anthropic manipulation of the species (e.g., domestication and cultivation) would amplify the genetic variability of this nuclear molecular marker. Thus, ITS2 has been discarded from the analyses of the tea samples in the present study, reflecting potential double peaks and noise in the raw data (i.e., ambiguous sequences) and thus reflecting its intrinsic nature and not the presence of actual contaminations or adulterants. Indeed, for the simple characterization of the species, the consensus sequence could be used as reported in Lee *et al*. [55], even if careful manual editing must be performed to obtain the sequences.

Another molecular marker discarded as an ideal sequencing barcode was *trn*H^(GUG)^-*psb*A. Even if this marker is easily amplified, its sequence was not discriminant among congeneric *Camellia* species, in contrast to *rbc*L+*mat*K. Indeed, BLAST *trn*H^(GUG)^-*psb*A sequence comparison (excluding polyA_n_) showed 100% identity in addition to *C*. *sinensis*, with more than ten *Camellia* taxa [e.g., *C*. *cuspidata* (Kochs) H. J. Veitch, *C*. *danzaiensis* H.T.Chang & K.M.Lan, *C*. *elongata* (Rehder & E.H.Wilson) Rehder, *C*. *euryoides* Lindley, *C*. *fraterna* Hance, *C*. *forrestii* (Diels) Cohen-Stuart, *C*. *grandibracteata* Hung T. Chang & F. L. Yu, *C*. *leptophylla* S. Ye Liang ex Hung T. Chang, *C*. *oleifera* Abel, *C*. *pitardii* Cohen-Stuart, *C*. *pubicosta* Merr., *C*. *reticulata* Lindley and *C*. *yunnanensis* (Pitard ex Diels)]. Some of these taxa resulted in incomplete sequences, resulting in incorrect comparison outputs (e.g., *C*. *cuspidata*, *C*. *elongata*, *C*. *euryoides C*. *fraterna*, *C*. *forrestii*, *C*. *oleifera*, *C*. *reticulate*, and *C*. *yunnanensis*). Notably, a polyA_n_ sequence was detected in *trn*H^(GUG)^-*psb*A, representing a potentially diagnostic marker to discriminate species similar to *C*. *sinensis* (e.g., *C*. *sinensis* A_13_ vs. *C*. *pubicosta* A_14_) but showing the intrinsic problematic nature of a polyN (e.g., stuttering, homoplasy). Thus, this marker was not used for taxonomical discrimination in the present study.

In contrast, the *rbc*L marker was used, reflecting both good discrimination power and universal primer amplification [31] that can also detect the macro-contamination of other species [56]. This marker resulted in two haplotypes for a single nucleotide polymorphism {SNP-A and SNP-C (M)} in the 68 bp coding region of *rbc*L (5’-AAATTGA-M-TTATTA-3’, GenBank reference: KJ806281, 57209 bp; *C*. *sinensis* var. *sinensis*, complete chloroplast genome), consistent with the report of Stoeckle *et al*. [31]. Indeed, according to these authors, when geography, tea type and taxonomical characterizations were available for *C*. *sinensis* reference samples, this nucleotide variation (SNP-C vs. SNP-A) was strongly associated with products from India in comparison with China, with black vs. green tea and var. *assamica* vs. var. *sinensis*, respectively. Notably, based on the increase of new accessions in GenBank (e.g., new *Camellia* chloroplast genomes [57, 58, 59], currently the SNP-C shows a lower taxonomical discrimination level compared with SNP-A. In subsequent BLAST searches, haplotype SNP-A discriminates for *C*. *leptophylla*, *C*. *oleifera* and *C*. *sinensis*; in contrast, haplotype SNP-C is also associated with *C*. *sinensis*, *C*. *crapnelliana* Tutcher, *C*. *cuspidata*, *C*. *grandibracteata*, *C*. *granthamiana* Sealy (= *C*. *albogigas* Hu), *C*. *impressinervis* H.T.Chang & S.Y.Liang, *C*. *japonica* L., C. kissii Wall, *C*. *oleifera*, *C*. *petelotii* (Merr.) Sealy, *C*. *pubicosta*, *C*. *sasanqua* Thunb., *C*. *taliensis* (W. W. Sm.) Melch., *C*. *tonkinensis* (Pit.) Cohen-Stuart and *Tutcheria hirta* (Hand.-Mazz.) H. L. Li (= *Pyrenaria hirta* (Hand.-Mazz.) H. Keng).

According to the *mat*K marker, even if its primers did not show good universality [31, 33, 34], this marker was used to confirm the presence of *C*. *sinensis* when *rbc*L results in SNP-A or to narrow the taxonomical field in the teabags when the SNP-C *rbc*L haplotype was present in the sample. BLAST analyses suggest that this barcoding molecular marker was able to discriminate the four *Camellia* species (*C*. *grandibracteata*, *C*. *leptophylla*, *C*. *pubicosta* and *C*. *sinensis*) with 100% identity.

Based on these results, an additional and exclusive marker has been demonstrated to discriminate *C*. *pubicosta* from *C*. *sinensis*, when the templates represent SNP-C in *rbc*L, as suggested above. Through the alignment of the chloroplast genomes of these two species, the intergenic region between *rps*7 and *trn*V^(GAC)^ was selected for the presence of two exclusive deletions in *C*. *pubicosta* (7 and 6 bp; 102651 and 102718 bp, respectively; GenBank reference: KJ806277, *C*. *pubicosta*, complete chloroplast genome). The oligos were designed between these two deletions, amplifying a 226 bp fragment in *C*. *pubicosta* and a 239 bp fragment in *C*. *sinensis* for both sequencing and genotyping analyses (Table 2).

#### PCR capillary genotyping

According to capillary genotyping electropherograms, ITS2 was excluded due to the presence of multiple peaks, as confirmed by previous sequencing results. Even if the *trn*H^(GUG)^-*psb*A intergenic spacer showed a nucleotide sequence shared with other allied *Camellia* species, this molecular marker was demonstrated as a better molecular marker than the P6 loop. In fact, *trn*H^(GUG)^-*psb*A, when compared with the P6 loop, showed a better discriminant power in terms of length (e.g., in eudicotyledons, the *trn*H^(GUG)^-*psb*A range is from 152 bp to 851 bp [60]; and the P6 loop range varied from 22 bp to 122 bp, [61]), and nucleotide variability [39, 60, 61]. Surely, the P6 loop can be useful with a higher concentration of degraded DNA (i.e., ancient DNA), where it is difficult to obtain fragments longer than 150 bp [39, 62].

In conclusion, *trn*H^(GUG)^-*psb*A was employed as an important pre-screening marker to detect potential adulterants via genotyping, as this marker can individuate the polymorphisms of PCR as fragments of different lengths (observable as anomalous peaks) compared to the reference standard of *C*. *sinensis* and/or allied BLAST species, which result in the same length in bp (CS_TP_-ref = 510 bp). In parallel, *rps*7-*trn*V^(GAC)^ was also used to discriminate *C*. *pubicosta* (226 bp) from *C*. *sinensis* (239 bp) for the difference in length of the amplified fragment (see above in paragraph “PCR sequencing”).

In the tea templates with only *C*. *sinensis* certified on the label (Table 1), the anomalous peaks with an RFU < 5%, (compared to the peak of *C*. *sinensis*) were interpreted as casual micro-contaminations and therefore excluded. Any deviation from the standard (i.e., reference profile of the listed ingredients) has been further investigated through cloning and sequencing the anomalous fragments.

### DNA Verity Test (DVT) applied to commercial teabags

#### DNA extraction (Exgene Plant SV kit)

All DNA extracted from tea samples showed a degraded pattern on electrophoretic analysis; lower concentrations were observed in black tea and in decaffeinated tea samples (N11, N15 and V7). In these templates, electrophoretic analysis showed a more degraded DNA genomic pattern than the others. No DNA was detected in soluble tea samples (N16 and V16).

#### PCR amplification {*trn*H^(GUG)^-*psb*A and *rps*7-*trn*V^(GAC)^ fluorescently labeled; *rbc*L and *mat*K}

No amplification of any tested marker was obtained from the two soluble tea samples (N16 and V16). Cases of recalcitrant PCR amplifications were observed for all decaffeinated samples (V7, N7, N11 and N15) and some black tea samples (N1, N4, N7, N11, N15). These recalcitrant DNA samples were amplified using AmpONE Fast Pfu DNA Polymerase, which was more robust than Phusion DNA Polymerase.

#### PCR genotyping {*trn*H^(GUG)^-*psb*A and *rps*7-*trn*V^(GAC)^ fluorescently labeled}

Using the genotyping of *trn*H^(GUG)^-*psb*A for pre-screening, all the samples showed a peak at 510 bp, corresponding to the *C*. *sinensis* reference (CS_TP_-ref). However, several samples were characterized based on anomalous peaks. For example, among the teabags that listed only *C*. *sinensis*, a black decaffeinated sample (N11) resulted in the presence of an additional, highly visible peak at 365 bp {RFU(365bp:510bp) = ca. 1:2}; in contrast, in the tea samples with additional material from other plants (admixture teas, V9-V11), the capillary genotyping data did not correspond with the number and intensity of peaks deduced by ingredients certified on the label, except for the confirmed presence of *C*. *sinensis* (peak at 510 bp). For example, V10 presents only an additional peak, which could be due to anise {ca. 317 bp; RFU (317 bp:510 bp) = ca. 1:3}, while the peaks of other certified ingredients, namely cinnamon (523 bp) and licorice (433 bp), were not detected. Low peaks (0.5% < RFU < 1.2%), assume to be from *Citrus* species (lemon, orange, grapefruit, and lime, ca. 552–577 bp) were individuated in the V9 green tea sample. A similar pattern was observed in the V11 sample (green tea), where a peak corresponding to mint (*Mentha* sp.) (ca. 436 bp) showed an RFU of approximately 1.7%, as referenced to the *C*. *sinensis* peak {i.e., RFU (436 bp:510 bp) = ca. 1:57}.

As a consequence of these unexpected results, two further procedures were used: (1) the *trn*H^(GUG)^-*psb*A of samples with anomalous peaks numbers was sequenced in addition and successively cloned; and (2) the admixture samples (V9, V10 and V11) with a lower number or less marked peaks compared with the reference profiles of the listed ingredients were amplified using the primers for the *trn*H^(GUG)^-*psb*A specifically designed in the present study (Table 2) (see paragraph, “Anomalous tea template”). The *trn*H^(GUG)^-*psb*A marker was also sequenced in the samples with normal capillary genotyping data (one peak at 510 bp) to generate reference Sanger chromatograms for comparison and no variability was observed among the accessions (GenBank accession: KY989996).

In addition, the genotyping of *rps*7-*trn*V^(GAC)^ can be a suitable additional pre-screening short marker for eventual contaminants, even if this molecular region is not yet well represented in GenBank; in contrast, it has been useful to discriminate between *C*. *sinensis* and *C*. *pubicosta*. The capillary genotyping patterns confirmed the results obtained by the sequencing approach (see results in the next paragraph). Surely, the genotyping approach was faster and more efficient compared with Sanger sequencing.

#### PCR sequencing {*rbc*L+*mat*K+*rps*7-*trn*V^(GAC)^}

According to the chromatograms of the *rbc*L sequences, the tea samples showed the presence of the two haplotypes (SNP-A and SNP-C; GenBank accession: KY989997 and KY989998, respectively) (Table 1), as also reported by Stoeckle *et al*. [31]. In the templates analyzed in the present study, among the green teas, six samples showed the presence of SNP-A, one sample indicated the presence of SNP-C and four showed heterozygous peaks (SNP-M), with adenine more represented than cytosine (V5 and V12, SNP-A/c) or similarly represented (V14 and V15, SNP-A/C). All the black tea samples showed the presence of SNP-C, except for five accessions, characterized by a heterozygous peak (SNP-M) (Table 1), with cytosine more represented than adenine (N5, N9, N11 and N13, SNP-C/a) and vice versa (N1, SNP-A/c). An additional oligo (CS_rbcL-300rev) was designed as an additional assessment of this SNP (Table 2).

In the *rbc*L sequences of the samples with additional peaks in the *trn*H^(GUG)^-*psb*A genotyping profile, the corresponding Sanger *rbc*L sequences resulted in multiple peaks (N11, V10) or decreased background noise (V9, V11).

Regarding the *mat*K sequences, no variability was observed among the tea accessions (GenBank accession: KY989999), and no multiple peaks were evident for the anomalous and/or admixture templates as in the corresponding *rbc*L sequences, confirming the low universality of these primers to detect potential DNA contaminations or different DNA in the admixture tea templates (e.g., [34]).

*Rps*7-*trn*V^(GAC)^ was amplified and sequenced to discriminate *C*. *sinensis* (haplo-CS 239 bp; GenBank accession: KY990000) from *C*. *pubicosta* (haplo-CP 226 bp; GenBank accession: KY990001) in tea templates where the SNP-C haplotypes of *rbc*L were present. This marker was also sequenced in samples with SNP-A haplotypes of *rbc*L to generate reference Sanger chromatograms for comparison.

Seven black templates (N1-N5, N7 and N15) revealed the presence of *rps*7-*trn*V^(GAC)^ belonging to *C*. *sinensis;* in contrast, several tea templates showed a sequence with multiple peaks, initiated by the first indel present in *C*. *sinensis* vs. *C*. *pubicosta* alignment. In some templates, the sequences were unreadable on account of the same height of the peaks (V9, V10, V11 and N9), but in the other ones, the sequence most represented can be deduced (V6, V8, N6, N8, N10-N14) (Table 1). In the latter case, the most represented sequence was easily analyzed and corresponded to that of *C*. *sinensis*. For the templates with peaks of the same intensity, a 2% agarose gel electrophoresis showed clear separation of the two bands; in contrast, in the other templates, the PCR was cloned and sequenced. The analyses of Sanger electropherograms applied to these fragments confirmed the presence of both *C*. *pubicosta* and *C*. *sinensis* in the same amplified templates (Table 1). As anticipated in the previous paragraph, these data were confirmed using a genotyping approach.

#### Anomalous tea template

According to the previous capillary genotyping data, the following two typologies of anomalous tea templates occur in our sampling: (I) (simple) sample with additional peak(s), even if *C*. *sinensis* was certified as the only ingredient (N11) and (II) admixture samples with the number or height of the peaks lower than expected in terms of species listed as ingredients (V9, V10 and V11).

In the simple tea templates (i.e., point I), N11 (a black decaffeinated sample) showed the presence of *trn*H^(GUG)^-*psb*A Sanger-sequences with additional peaks. The sequences of *trn*H^(GUG)^-*psb*A clones of the additional peak were identical (GenBank accession: KY928308). The best identity (query cover) and E-value obtained were, respectively, only 84% (100%) and 8e-^86^, which was discriminatory for *Pipturus argenteus* (G. Forst.) Wedd. (Urticaceae, Boehmerieae) (GenBank accession: KU564710). These low values reflected the absence of an exhaustive data bank for this molecular marker. Thus, we also cloned the *rbc*L sequence, where multiple peaks were evident (see above). The obtained *rbc*L sequence of the contaminant (GenBank accession: KY928310) best matches with *Pouzolzia* sp. [585/585 bp (100%), query cover = 96%, E-value = 0, GenBank accession: KF138239) and namely with two accessions of *P*. *zeylanica* (L.) Benn. [583/585 bp (99.5%), query cover = 96%, E-value = 0, GenBank accession: KF138241; and 552/552 bp (100%), query cover = 91%, E-value = 0, GenBank accession: KF496389]. According to these results, we hypothesized the contaminant was a tropical taxon belonging to Urticaceae family and was likely a *Pouzolzia* taxon. Briefly, *Pouzolzia* Gaudich. (Urticaceae, Boehmerieae) is a pantropical genus of shrubs or herbs, [63, 64] represented by approximately 40 species [65, 66, 67, 68, 69] (only 32 species were assessed using The Plant List [70]). Currently, only the following six species are present in GenBank based on *rbc*L sequences: *P*. *australis* (Endl.) Friis & Wilmot-Dear, recently transferred from *Boehmeria* Jacq. [71], *P*. *calophylla* W.T. Wang & C.J. Chen (by the synonym *P*. *argenteonitida* W.T. Wang), *P*. *guineensis* Benth., *P*. *sanguinea* (Blume) Merr. (within var. *sanguine* and var. *elegans* [Wedd.] Friis & Wilmot-Dear), *P*. *mixta* Solms, and *P*. *zeylanica*. Moreover, *mat*K sequences are listed for only the following four species: *P*. *guineensis*, *P*. *mixta*, *P*. *sanguinea* (both for var. *sanguinea* and var. *elegans*), and *P*. *zeylanica*, indicating incomplete DNA barcoding characterization. *Pouzolzia* species are well known in the Old Tropical World as sources of fiber [63] and for their medicinal proprieties. For example, *P*. *zeylanica* (= *P*. *indica* Linn.) is also commonly used as an officinal plant (root, steam and leaves) for some medical remedies (e.g., mastitis, pyogenic infection, diarrhea, indigestion, and vulnerary) [72, 73, 74]. Moreover, *P*. *hirta* (L.) Hassk. var. *hirta* (= *P*. *bennettiana* Wight) is largely employed as a medicinal brew, referred to as “*Oyik*” in India [75]. *Pouzolzia zeylanica* or allied taxa are frequent in the area where *C*. *sinensis* is cultivated [63], explaining the remarkable presence of this plant (approximately 50%) as a filler in the black tea (N11) examined in this study.

In the admixture templates (i.e., point II), V10 had an obvious contaminated sequence, but only one certified additional ingredient was identified as anise {*trn*H^(GUG)^-*psb*A clones = *Pimpinella anisum/aromatic/flabellifolia* 100% identity; GenBank accession: KY928309}, while the other two are lacking (cinnamon and licorice). In contrast, V9 and V11 showed lower additional peaks at ca. 430 bp for *Mentha* sp. and ca. 552–577 bp for *Citrus* sp., respectively. These admixture templates were amplified using specific primers for the plant species identified in the ingredient list but were not clearly assessed in the genotyping and sequencing/cloning data.

According to the PCR results, in the template V10, no amplification fragments were obtained using the primers specific for cinnamon and licorice, confirming the absence of these two certified ingredients. According to the amplification and cloning results, anise was well represented in this tea green template and did not correspond with the quantity reported on the labels (2%). Considering the two other green templates, V9 and V11, positive amplifications were obtained using the primers specific for *Citrus* and *Mentha* sp., respectively, confirming the presence of these plants but not in the quantity listed on these green tea templates (i.e., *Citrus* 2% and *Mentha* 7.9% in V9 and V11, respectively).

#### Taxonomical characterization and systematic considerations

The level of specific discrimination was optimal, confirming both the presence of *C*. *sinensis* in the tea templates through the joint use of *rbc*L+*mat*K±*rps*7-*trn*V^(GAC)^ molecular markers. *Rps*7-*trn*V^(GAC)^ is an additional and exclusive marker, which has been fundamental to discriminate *C*. *sinensis* from *C*. *pubicosta*, where the *rbc*L sequence has the nucleotide cytosine at 68 bp (SNP-C) (Table 1).

In summary, *rbc*L(SNP-A)+*mat*K sequences can identify *C*. *sinensis* and *C*. *leptophylla*; in contrast, *rbc*L(SNP-C)+*mat*K+*rps*7-*trn*V^(GAC)^ matches *C*. *sinensis* and *C*. *grandibracteata*. This species and *C*. *leptophylla* are critical taxa, as these species are also regarded as mere varieties of *C*. *sinensis* or as not distinct from it and, thus, as potentially only two wild crop relatives of the cultivated tea [76, 77]. Moreover, both *C*. *leptophylla* and *C*. *grandibracteata* were characterized at an obscure or limited native range. Indeed, *C*. *leptophylla* has been identified only at the top of Daqing Mountain (Guangxi, western China) [78], while pertinent herbarium specimens from two further localities in Guangxi were subsequently identified as *C*. *sinensis* [77]. In addition, *C*. *grandibracteata* is exclusively identified from tea gardens along the Mekong River in Yunxian (south-west China) [76], and it would not seem to exist in the wild. Therefore, this species might result from spontaneous hybridization or introgression among cultivated plants (Shi-Xiong Yang and Dong-Wei Zhao, personal communication in [76]).

Based on the presence of the heterozygosis peaks in the *rbc*L sequence (SNP-A/C) and both fragments of the *rps*7-*trn*V^(GAC)^ sequence (haplo-CS 239 bp/haplo-CP 226 bp) (see Table 1), the leaf matrices present in some teabags could comprise the following: (1) an admixture of two haplotypes belonging to C. *sinensis* (potentially ascribable to var. *sinensis* and var. *assamica*, see [31]), (2) a mixture of *C*. *sinensis* and *C*. *pubicosta*, or (3) an admixture of *C*. *sinensis* and hybrids of *C*. *pubicosta* (♀)/*C*. *sinensis* (♂). These speculations are based on the presence of maternal inheritance in the chloroplast of *C*. *sinensis* according to Kaundun and Matsumoto [79].

In detail, *C*. *pubicosta* is a critical species, strictly allied to *C*. *sinensis*. The first specimens collected from Mount Bavi (currently in Vietnam) were tentatively included in *C*. *sinensis*. However, later, Merrill [80] recognized a new species by analyzing additional material, distinct from *C*. *sinensis* on account of relevant features in his opinion. Indeed, some of the vegetative features reported in the protologue [80] could be included in the variability of *C*. *sinensis*. For example, the acuminate-caudate leaves also occur in *C*. *sinensis* var. *assamica*. However, *C*. *pubicosta* appears distinct from *C*. *sinensis* s.l. based on other relevant traits, chiefly due to its three styles, which are free and not connate as in *C*. *sinensis*. Thus, *C*. *pubicosta* was placed in the sect. *Corallinae* Sealy, and *C*. *sinensis* belongs to the sect. *Thea* (L.) Benth. & Hook. [81]. However, this point is controversial, as other scholars [82], in contrast, also include *C*. *pubicosta* in the sect. *Thea*. According to the recent chloroplast genome phylogeny of Huang *et al*. [59], the authors likely put an end to the dispute, as based on their analysis, *C*. *pubicosta* was identified as a sister group to both *C*. *sinensis* var. *assamica* and *C*. *grandibracteata* terminal and therefore is better placed in the sect. *Thea*. According to Huang *et al*. [59], the character of connate vs. free styles was not consistent. Finally, considering the phylogenetic distance of the investigated taxa in *Camellia* (see, *C*. *sinensis* var. *assamica* vs. *C*. *sinensis* var. *sinensis*), some species falling in the *C*. *sinensis* varieties clade included *C*. *pubicosta* and *C*. *grandibracteata* (cfr. Fig 9 [59]) but as further varieties and not different species.

## Conclusion

After packaging, the species from which the tea samples are obtained are morphologically unrecognizable. Therefore, we cannot know with certainty using traditional methods if the source plants are actually those reported in the listed ingredients. For this reason, in the present study, a standardized protocol of DNA barcoding characterization (*DNA Verity Test*, *DVT*) was developed to increase both efficiency and rapidity and to become current with the new molecular data submitted to GenBank. After the optimization of *the DVT* protocol (extraction efficiency, DNA polymerase fidelity, reliability amplification and the selection of barcoding markers), the 32 green/black tea samples of *C*. *sinensis* were analyzed in a blind test. The *DVT* protocol comprises two approaches used jointly, i.e., (1) a rapid pre-screening analysis through a genotyping approach using *trn*H^(GUG)^-*psb*A and *rps*7-*trn*V^(GAC)^ for individuating any trace of contamination by other plants (revealed by anomalous peaks in the capillary row data) and (2) a Sanger sequencing approach using the *rbc*L+*mat*K±*rps*7-*trn*V^(GAC)^ to confirm both the presence of *C*. *sinensis* and identify food frauds (i.e., contamination, absence of certified ingredients).

We can summarize some of the main results as follows:

1. The *DVT* protocol is suitable for detecting adulteration in tea matrices (contamination or the absence of certified ingredients) and can be applied to study other similar systems.
2. Using BLAST analysis of the sequences of *rbc*L+*mat*K±*rps*7-*trn*V^(GAC)^ chloroplast markers, *C*. *sinensis* can be taxonomically characterized.
3. *Rps*7-*trn*V^(GAC)^ can be employed to discriminate *C*. *sinensis* from *C*. *pubicosta*.
4. ITS2 is not an ideal DNA barcode for tea samples because of potential incomplete lineage sorting and hybridization/introgression phenomena in *C*. *sinensis* taxa.
5. This genotyping approach is an easy, inexpensive and rapid pre-screening method to detect anomalies in the tea templates.
6. Two herbal companies provided no authentic products with a contaminant (N11) or without some of the listed ingredients (V10).
7. The leaf matrices present in some teabags could comprise an admixture of different *C*. *sinensis* haplotypes and/or allied species (*C*. *pubicosta*).
