# Correction: What is in your cup of tea? *DNA Verity Test* to characterize black and green commercial teas

**DOI:** 10.1371/journal.pone.0192334

**Published:** 2018-01-30

**Authors:** Olga De Castro, Maria Comparone, Antonietta Di Maio, Emanuele Del Guacchio, Bruno Menale, Jacopo Troisi, Francesco Aliberti, Marco Trifuoggi, Marco Guida

In Table 2, the names and sequences listed for Cinnamon_2psb-for and Cinnamon_2trnH-rev are incorrect. The sequence listed for trnHf is also incorrect. Please see the corrected Table 2 here.

**Table 2:** Primers used in the present study for DNA barcoding analyses.

Locus	Primer name	Sequence (5’-3’)	Ta	Primer note	Reference
***rbc*L**	rbcLa_F	ATG TCA CCA CAA ACA GAG ACT AAA GC	55	Universal	[35]
	rbcLajf634R	GAA ACG GTC TCT CCA ACG CAT		Universal	[36]
	CS_rbcL-300rev	TAA AGG ATA CGC TAC ATA AGC	55	Specific for *Camellia sinensis*; used to check the SNP-M (68 bp) in sequencing PCR	In the present study
***mat*K**	1R_KIM-f	ACC CAG TCC ATC TGG AAA TCT TGG TTC	55	Universal	Ki-Joong Kim, pers. comm.
	3F_KIM-r	CGT ACA GTA CTT TTG TGT TTA CGA G			
***trn*H**^**(GUG)**^**-*psb*A**	psbA3'f	GTT ATG CAT GAA CGT AAT GCT C	55	Universal	[37]
	trnHf*	CGC GCA TGG TGG ATT CAC AAT CC			[38]
	Can_3psb-for	GTT TCT TCT GTA CCA ACC TGC	56	Specific for *Cinnamomum verum* and *C*. *cassia* (cinnamon)	In this study
	Can_3trnH-rev	TAC TTC ACT CTC CTT CCT GCT			
	Citrus_psbA-in	GTA TTG ATC CGT TAT TTA GTC G	57	Specific for *Citrus* sp. (lemon, orange, grapefruit, and lime)	In this study
	Citrus_trnH-in	ATC TTA TCT TAC TTA TGA AGA ACC			
	Glycyrrhiza_psbA-in	GTT TTA AAG AAG GAT ACG AGG	55	Specific for *Glycyrrhiza glabra* (licorice)	In this study
	Glycyrrhiza_trnH-inA	TAC ATT CGC CCT TCT TAT AC			
	Mentha_2psb_for	TTC CAG GCA AGT CCA ATA CT	56	Specific for *Mentha* sp. (*M*. *aquatica*, *M*. *arvensis*, *M*. *canadensis*, *M*. *longifolia*, *P*. *pulegium*, *M*. *suaveolens*, *M*. *pulegium*, and *M*. x *piperita*, *M*. *spicata*)	In this study
	Mentha_2trnH_rev	TGT GTA GGA GTT TTT GAA AAT AGA C			
	Piminella_psbA-in	ACC TAG CTG CTG TTG AAG CTC	60	Specific for *Pimpinella anisum* (anise)	In this study
	Pimpinella_trnH-in	GGA GCA ATA TCG CTT TCT TGA TAG A			
***rps*7-*trn*V**^**(GAC)**^	CS_*rps*7_1	GTG CTA TGG CTC GAA TCC GT	67	Specific for *Camellia* sp.; used to discriminate *C*. *sinensis* from *C*. *pubicosta*	In this study
	CS_*trn*V^(GAC)^_1*	ACC ACG TCA AGG TGA CAC TC			
**P6 loop *trn*L**^**(UAA)**^	g	GGG CAA TCC TGA GCC AA	55	Universal	[39]
	h*	CCA TTG AGT CTC TGC ACC TAT C			
**ITS2**	S2F	ATG CGA TAC TTG GTG TGA AT	55	Universal	[40]
	S3R*	GAC GCT TCT CCA GAC TAC AAT			
	BELL-1	GGD GCG GAK AHT GGC CYC CCG TGC	55	Universal	[41]
	BELL-3	GAC GCT TCT CCA GAC TAC AAT			

*, labeled
